# YOLOv13 Steel Surface Defect Detection Method Based on Multi-Scale Denoising Enhanced A2C2f Module

**DOI:** 10.3390/ma19102060

**Published:** 2026-05-14

**Authors:** Yang Meng, Bowen Yang, Fan Yang, Hua Li, Junzhou Huo

**Affiliations:** 1 School of Mechanical Engineering, Dalian University of Technology, Dalian 116024, China; menyang@mail.dlut.edu.cn (Y.M.); lihua0107@mail.dlut.edu.cn (H.L.); huojunzhou@dlut.edu.cn (J.H.); 2 School of Mechanical Engineering, Shenyang University of Technology, Shenyang 110870, China; 3 Norla Institute of Technical Physics, Chengdu 610211, China; youngfine_yang@163.com

**Keywords:** feature extraction module, backbone structure, deep neural network, steel defect detection

## Abstract

Steel surface quality critically determines the service safety and structural reliability of industrial products. Defects such as cracks, inclusions, patches, pitting, rolled-in scale, and scratches severely compromise product safety, making accurate and efficient detection a key step in quality control. However, the native A2C2f module in YOLOv13 exhibits insufficient multi-scale feature extraction for tiny defects and weak robustness under complex industrial backgrounds, hindering the detection of these six defect types. To address these gaps, we propose a multi-scale denoising enhanced module, A2C2f-MSDE, which constructs a multi-scale multi-kernel fusion branch (MSKF) with learnable adaptive weights, integrates a lightweight SEL channel attention and a DE denoising module, and employs a dual learnable residual scaling structure, while preserving the original multi-scale fusion architecture. We embed A2C2f-MSDE into the YOLOv13 backbone, perform ablation studies to verify each component’s contribution, compare it with mainstream advanced detectors on the public NEU-DET dataset, and conduct generalization tests on the GC10-DET dataset. Experiments on NEU-DET show that the improved YOLOv13n achieves mAP50-95 of 0.454 (9.4% relative gain over baseline, absolute gain 0.039), with mAP50 and mAP75 reaching 0.774 and 0.466, at an inference speed of 555 FPS, respectively, outperforming the compared mainstream models. On GC10-DET, mAP50 reaches 0.704, comparable to the baseline, maintaining stable overall detection capability, while mAP75 and mAP50-95 improve by 0.033 and 0.019, verifying the module’s performance advantages under high localization accuracy requirements and its cross-dataset generalization ability. The proposed module effectively balances detection accuracy and lightweight characteristics, providing a high-performance solution for industrial steel defect detection.

## 1. Introduction

Steel material serves as a fundamental core material in modern high-end equipment and industrial manufacturing, widely applied in aerospace, automotive manufacturing, bridge engineering, petrochemical industry, and mechanical equipment. Its surface quality directly determines whether end-user industrial products can meet the requirements for safe, reliable, and high-precision service. In the complex steel production and processing workflow, various surface defects such as cracks, inclusions, patches, pitting, oxide scales, and scratches inevitably occur on steel surfaces. In this work, surface defects are defined as anomalies that are exposed on the steel surface and can be visually detected, such as cracks, patches, pitted surfaces, rolled-in scale, and scratches. Subsurface defects lie just beneath the surface but are not visually exposed, while internal defects, such as non-metallic micro-inclusions, are located deeper within the material and are typically detected by volumetric non-destructive testing (NDT) techniques. It should be noted that the “inclusions” class in the NEU-DET dataset refers to surface-visible inclusion-type defects, not internal micro-inclusions, and is therefore considered a surface defect in this study. This paper focuses exclusively on the detection of surface defects using machine vision, and internal defects such as non-metallic micro-inclusions are beyond the scope of this work. These defects exhibit an extremely wide range of morphological scales, spanning from micro-scale micro-cracks to millimeter-scale patches and inclusions. In addition to morphological diversity, the final finishing process of steel products is directly related to the occurrence and detectability of surface defects. For example, peeled steels generally exhibit a lower defect rate than as-rolled steels, with reduced scale and surface irregularities. Conversely, rolling marks, corrosion, and scale on as-rolled surfaces are more prominent and comparatively easier to detect due to their distinct textural characteristics. The NEU-DET dataset employed in this study consists of hot-rolled strip steel, a typical as-rolled product. The prevalence of easily detectable defects, such as rolled-in scale and scratches, in this dataset aligns with the above analysis, providing a representative testbed for evaluating detection techniques under common industrial conditions. To prevent these surface defects from deteriorating the mechanical properties of steel, which could cause fatigue fracture, corrosion failure, or even catastrophic structural collapse during subsequent service, resulting in severe casualties and property losses, achieving timely, accurate, and automated detection and identification of steel surface defects has become a core requirement of the modern steel industry and a key research direction in the field of industrial visual inspection. Furthermore, the determination of probable surface defect types and an in-depth understanding of their morphological characteristics are essential for both detection quality and industrial decision-making. For instance, cracks typically appear as elongated, low-contrast lines, while patches and rolled-in scale exhibit larger-scale textural patterns. Such prior knowledge not only guides the development of more targeted feature extraction strategies but also helps operators interpret detection results and make informed decisions on product grading, repair, or rejection. It should be clarified that this study focuses exclusively on hot-rolled strip steel in the as-rolled condition, as represented by the NEU-DET dataset, and the six defect types investigated—cracks, surface-visible inclusions, patches, pitted surfaces, rolled-in scale, and scratches—are specifically validated in this context. Steel conditions such as forged, machined, peeled, or coated surfaces are beyond the scope of the present work. Furthermore, the proposed vision-based detection technique is intended as a screening aid for quality inspection rather than a fully autonomous decision-making system. Effective industrial deployment still requires significant operator training to interpret detection results in conjunction with metallurgical knowledge and make informed disposition decisions. Reliance on detection methods without sufficient operational maturity or qualified human oversight may result in overlooked critical defects and, in the worst case, catastrophic structural failures during service.

Traditional manual visual inspection methods for steel surface defects suffer from inherent limitations such as low detection efficiency, poor accuracy, and high subjectivity, making them unsuitable for the high-speed, continuous production rhythm of the modern steel industry. Although physical effect-driven non-destructive testing (NDT) techniques, such as low-cost magnetic flux leakage (MFL) testing based on Hall sensors [1], Gaussian window frequency modulated thermal wave imaging (GWFMTWI) [2], and magneto-acoustic emission (MAE) [3], can achieve satisfactory detection results for specific defect types, they generally face practical limitations and technical bottlenecks, including constrained detection scenarios, high equipment costs, poor repeatability, and difficult interpretation, making it challenging to meet the urgent demand for high-precision, full-process, automated detection in the modern steel industry. In contrast, machine vision-based steel surface defect detection technology, after decades of development and industrial deployment, has formed a complete system encompassing image acquisition hardware and full-process automated algorithms [4]. As a non-contact inspection solution, it can adapt to the inspection requirements of various steel types, offering advantages such as high precision, strong online adaptability, and ease of integration. Meanwhile, advancements in traditional image processing algorithms and the development of deep learning technologies have driven industrial inspection paradigms toward end-to-end intelligent detection. The successive introduction and continuous optimization of object detection models such as Faster R-CNN, SSD, and YOLO have effectively addressed the challenges of small samples and high real-time requirements in industrial scenarios, providing breakthrough technical support for intelligent steel surface defect detection.

It should be clarified that steel surface defects in industrial scenarios differ fundamentally from general object detection targets in natural scenes in terms of size distribution and morphological characteristics. In industrial captured images, steel surface defects exhibit extreme scale heterogeneity: large-scale defects such as patches and large-area inclusions may occupy the majority of the image, while tiny defects such as pitting, micro-cracks, and fine scratches account for an extremely low proportion of effective pixels. Moreover, defect contours are mostly irregular in shape and exhibit very low contrast compared with complex rolling background textures. This coexistence of extreme scale differences, irregular morphologies, and low signal-to-noise ratio (SNR) poses significant challenges to general-purpose object detection models.

To address the above industrial challenges and technical limitations, this paper takes the latest YOLOv13 object detection model as the baseline, with the core goal of substantially improving feature learning for steel surface defects, and focuses on optimizing the feature extraction module in the backbone network. The main research objectives of this paper are: (1) to design a multi-scale multi-kernel fusion branch with learnable adaptive weights that can simultaneously capture local details of tiny defects and global semantics of large-scale defects; (2) to integrate a lightweight channel attention module and a denoising enhancement module to suppress complex background noise and strengthen edge textures, thereby improving robustness in industrial scenarios; (3) to introduce a dual learnable residual scaling structure to preserve gradient flow integrity and enhance training stability; and (4) to validate the effectiveness and generalization ability of the proposed module through comprehensive ablation studies, comparisons with mainstream advanced detectors, and cross-dataset tests.

In pursuit of these objectives, the following core research work was carried out: (1) A novel multi-scale denoising enhanced module, A2C2f-MSDE, was designed to capture and hierarchically fuse defect features at different scales, effectively mitigating feature attenuation of tiny defects and improving multi-scale feature extraction efficiency. (2) A multi-scale multi-kernel fusion branch, MSKF, with learnable adaptive weights was constructed, adopting a dual-branch parallel convolution structure and embedding a lightweight SEL channel attention module to achieve adaptive channel-wise feature weighting, thereby enhancing the response of effective defect features and suppressing background noise. (3) A dedicated DE denoising enhancement module was cascaded to further purify defect features and enhance edge textures, improving the model’s discriminative ability for low-contrast defects under complex backgrounds. (4) The residual structure and training stability were optimized by introducing a dual learnable residual scaling coefficient in the A2C2f-MSDE module, effectively preserving gradient flow integrity in deep networks, alleviating the vanishing gradient problem, and improving feature reuse capability and training convergence stability. Comprehensive comparative experiments were conducted on the public NEU-DET steel surface defect dataset released by Northeastern University, and generalization tests were performed on the public GC10-DET steel surface defect dataset.

## 2. Related Works

The feature extraction module in the backbone network serves as the core foundational component in the YOLO series detection models, bridging the input image with subsequent feature fusion and the detection head. It undertakes two primary functions: first, extracting discriminative target features from the input image, and second, providing high-quality feature representations for the subsequent neck feature fusion and detection head regression. A typical feature extraction module consists of convolutional layers, residual structures, attention mechanisms, and specialized feature enhancement components. This architecture effectively captures shallow spatial details and deep semantic abstraction information of targets, significantly improving model detection performance and robustness, which is particularly crucial for multi-scale object detection tasks involving tiny industrial defects. In recent years, the design methodology for core feature extraction modules in the YOLO series has been continuously evolving and optimizing.

CSPNet, proposed by Wang et al. [5], effectively addresses the issue of redundant gradient information learning during network optimization through cross-stage partial connections and feature “split-transform-merge” operations, achieving more efficient feature extraction. This provided a paradigm for subsequent backbone network designs in the YOLO series, which first introduced the CSPNet concept in YOLOv4 [6] to construct the CSPDarknet53 backbone. Building upon this, the C2f module proposed in YOLOv8 designs richer gradient flow paths through a dual-branch residual structure and cross-layer feature concatenation, significantly enhancing feature extraction capability while balancing computational efficiency, becoming the core feature extraction module for YOLOv8 and YOLOv10 [7]. YOLOv11 [8], on the other hand, adopts a modified C3k2 module to replace C2f as the core computational unit in its backbone and neck networks. YOLOv12 [9] constructs an attention-centric architecture, with its core innovations being the A2 (Area Attention) module and R-ELAN (Residual Efficient Layer Aggregation Network). The A2 module achieves self-attention computation within local windows through a minimalist feature partitioning strategy, while R-ELAN addresses the model optimization challenges introduced by attention modules through block-level residual design and reorganized feature aggregation, ultimately achieving a better balance between feature extraction capability and computational cost. In feature enhancement research, the SE module proposed by Hu et al. [10] pioneered the channel attention mechanism, achieving adaptive weighting of channel features through global information embedding and effectively enhancing the feature response of valid targets. The CBAM module proposed by Woo et al. [11] further integrates a spatial attention branch on top of channel attention, realizing adaptive feature enhancement in both channel and spatial dimensions.

At the engineering application level, optimization of feature extraction modules has achieved significant research progress in the field of steel surface defect detection. Wang et al. [12] designed the CGSF feature extraction module and lightweight SPPF-DW module, which decouple spatial and channel feature extraction through backbone output reuse and depthwise separable convolutions, significantly enhancing the capture and retention of both shallow and deep defect features on steel surfaces while reducing computational complexity. The research team of Pan et al. [13] constructed a CNN-Transformer dual-encoder backbone network, achieving simultaneous precise capture of local defect texture details and complete modeling of global semantic context through a dual-path architecture combining adaptive kernel convolution (AKConv) and Vision Transformer, while also designing the C2f_EEM edge enhancement module to further strengthen the extraction of defect contour and boundary features. Li et al. [14] introduced deformable convolution (DCN) to reconstruct the C2f module of YOLOv8, proposing the C2f_DCN feature extraction module, which achieves adaptive learning and representation of steel defect features of varying sizes and irregular shapes through a deformable sampling mechanism, while embedding an explicit visual center (EVC) module to enhance the model’s adaptive adjustment capability for features across different levels and scales. Wang et al. [15] developed the C3-EI edge-enhanced feature extraction module, which integrates a Sobel edge convolution branch with a standard CNN branch to construct a dual-path feature extraction architecture, combined with a deformable attention Transformer mechanism, enabling precise extraction of edge features from subtle defects such as cracks and scratches on steel surfaces and dynamic focusing on key regions. Zeng et al. [16] proposed the C2f_MLCA module, incorporating mixed local channel attention, which effectively addresses the issue of feature information loss caused by the limited receptive field of the traditional C2f module by integrating local and global, channel and spatial attention information, while pairing it with the GSConv lightweight convolution module to reduce computational overhead in feature extraction while minimizing information loss during channel transformation. Zhao et al. [17] reconstructed the backbone feature extraction network based on the FasterNet architecture and PConv convolution, significantly reducing computational redundancy and memory access during feature extraction, while designing the C2f-GAM global attention module and SK selective kernel attention module to achieve precise focusing and efficient extraction of steel defect features under complex backgrounds through dynamic feature response adjustment and adaptive receptive field optimization.

Overall, current optimization strategies for feature extraction modules in steel surface defect detection mainly focus on three major directions: (1) Lightweight and adaptive convolution architecture innovation, which achieves adaptive feature capture for defects of varying morphologies, scales, and irregular shapes while controlling model computational complexity and parameter count through the design and application of novel convolution operators such as depthwise separable convolution, deformable convolution, and partial convolution; (2) Multi-dimensional attention mechanism fusion optimization, which achieves adaptive weighting of discriminative defect features and effective suppression of complex industrial background noise through the introduction of mechanisms such as channel-spatial joint attention, global context attention, and dynamic gated attention, while enhancing the perception of tiny defects and edge contour features; (3) Multi-branch and multi-modal feature fusion architecture design, which compensates for the representational shortcomings of single feature extraction architectures and achieves comprehensive complementary representation of local defect details and global semantics by constructing fusion structures such as CNN-Transformer dual encoders, edge feature-semantic feature dual branches, and cross-level reuse of shallow and deep features.

Although existing methods can achieve satisfactory detection results under specific working conditions, there remain unresolved common limitations in terms of feature preservation capability for tiny, low-contrast defects on steel surfaces, anti-interference robustness in complex industrial backgrounds, and the ultimate balance between detection accuracy and computational efficiency required for edge-side deployment. To address these unresolved challenges, this paper proposes a novel A2C2f-MSDE feature extraction module, which systematically optimizes around adaptive fusion of multi-scale defect features, industrial scene noise suppression, gradient flow optimization, and deep feature reuse mechanisms.

## 3. Materials and Methods

### 3.1. Overall Architecture

YOLOv13 is the latest single-stage object detection model in the YOLO series. Balancing detection accuracy and inference speed, it achieves significant improvements in multi-scale object detection performance in complex scenarios through core innovations such as hypergraph adaptive association enhancement and full-pipeline feature aggregation allocation [18]. Its overall network architecture follows the classic Input-Backbone-Neck-Head four-stage structure of the YOLO series.

Within this architecture, the Backbone serves as the feature extraction core of YOLOv13, responsible for progressively extracting shallow texture details and deep semantic features from the input image. The resulting P3 to P5 feature maps directly determine the upper bound of accuracy for subsequent Neck fusion and detection head recognition. The Backbone employs the DSC3k2 module, optimized with depthwise separable convolution (DSConv), and the A2C2f module as its core feature extraction units, utilizing hierarchical downsampling and residual connections to enhance feature representation capability while reducing parameter count. When the feature extraction capability of the backbone network is insufficient, the detailed features of tiny, low-contrast defects may severely attenuate or even vanish during progressive downsampling, thereby hindering the effective fusion of shallow geometric details and deep semantic features in the Neck stage. Furthermore, under complex industrial backgrounds, if the feature extraction module lacks discriminative feature weighting and noise suppression mechanisms, it becomes highly susceptible to confusion between actual defects and similar background textures. Both scenarios can lead to a significant degradation in the performance of deep detection models for steel surface defect detection tasks. Therefore, the improvement in this paper focuses on the A2C2f module within the Backbone, aiming to enhance feature extraction capability for tiny defects on steel surfaces, mitigate feature attenuation and background interference, and achieve accurate detection of small defect targets.

In the backbone network of this model, the A2C2f module serves as the fundamental unit for core feature extraction. The A2C2f-MSDE module proposed in this paper can fully replace the original A2C2f module. To maximize the extraction of discriminative features from steel surface defects across different scales, the backbone network integrates two core optimization components:

(1) Multi-scale denoising enhanced A2C2f cascade module: The cascaded A2C2f-MSDE modules form the core mechanism for achieving hierarchical feature extraction and enhancement in the model;
(2) Optimized residual structure with dual learnable scaling coefficients: This structure connects residual layers both within and between modules, mitigating feature degradation while enhancing the stability of gradient propagation.

### 3.2. Multi-Scale Denoising Enhanced A2C2f-MSDE Module

**Figure 1:**
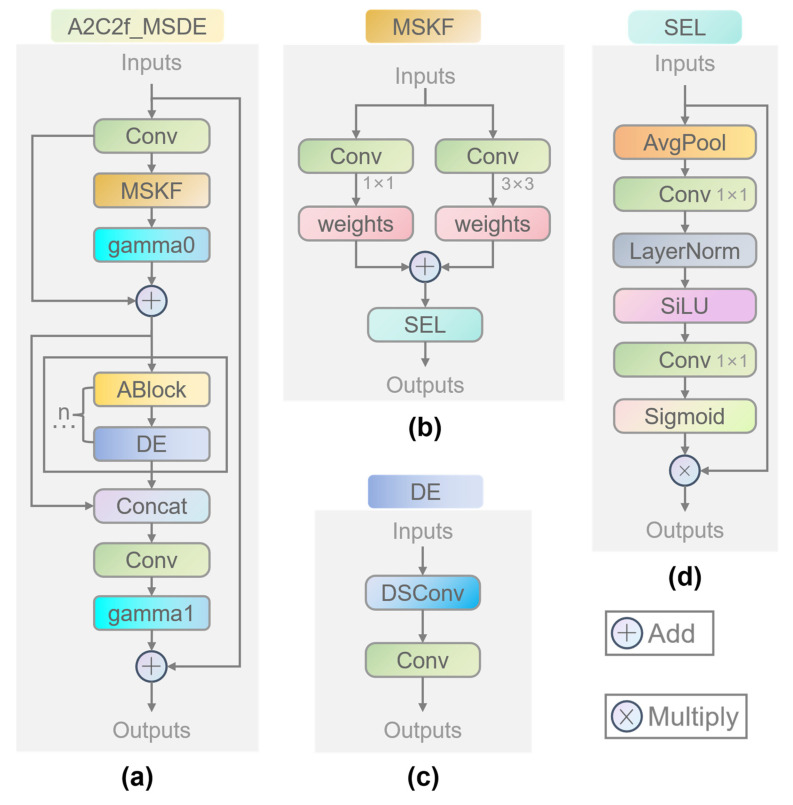
Architecture of the proposed improved modules. (**a**) Structure of the multi-scale denoising enhanced A2C2f-MSDE module, n represents that there are n combinations of ABlock modules and DE modules, and the number n can be specified in the model; (**b**) Structure of the MSKF sub-module: multi-scale multi-kernel fusion branch; (**c**) Structure of the DE sub-module: denoising and texture enhancement module; (**d**) Structure of the SEL sub-module: lightweight channel attention module.

The original A2C2f module performs excellently in general object detection tasks, but exhibits three major limitations in the specific industrial scenario of steel surface defect detection. First, the scale span of steel defects is enormous, ranging from micro-scale micro-cracks to millimeter-scale patches, making it difficult for the original module’s multi-scale feature extraction capability to simultaneously adapt to both extremes. Second, the coexistence of complex rolling background textures and low-contrast defect features, combined with the lack of dedicated noise suppression and edge enhancement mechanisms, makes the module susceptible to misclassifying background interference as defects. Third, as network depth increases, the vanishing gradient problem gradually emerges, affecting effective learning of deep features and the training convergence stability of the module. To address the above issues, this paper designs the A2C2f-MSDE module, whose structure is shown in Figure 1a. Through the synergistic effect of four major mechanisms—multi-scale fusion, attention weighting, denoising enhancement, and a residual optimization structure with dual learnable scaling coefficients—this module achieves efficient extraction and robust representation of steel defect features.

In the first stage of the A2C2f-MSDE module, input channel dimension transformation and preliminary multi-scale feature fusion are performed. The input feature map x first passes through a 1×1 convolution (cv1) to transform the channel dimension, producing a feature map $x_{1}$ with a hidden channel dimension, as shown in Equation (1). Subsequently, $x_{1}$ is fed into the MSKF multi-scale multi-kernel fusion branch, which fuses features from the 1×1 convolution branch and the 3×3 convolution branch via learnable adaptive weights, followed by feature enhancement through the lightweight SEL channel attention module, outputting a multi-scale enhanced feature map $x_{\mathrm{msf}}$. A residual fusion of $x_{1}$ and $x_{\mathrm{msf}}$ is then performed using the learnable scaling coefficient $\gamma_{\mathrm{msf}}$, as shown in Equation (2), which preserves the original feature information while enhancing the multi-scale feature representation.

(1) $$x_{1}={\mathrm{Conv}}_{1\times 1}\left(x\right)$$

(2) $$x_{\mathrm{msf}\_\mathrm{out}}=x_{1}+\gamma_{\mathrm{msf}}\cdot \mathrm{MSKF}(x_{1})$$

In the second stage of the module, deep feature extraction and denoising enhancement are performed on $x_{\mathrm{msf}\_\mathrm{out}}$. The feature map is sequentially processed by n cascaded modules, each consisting of an ABlock region attention module followed by a DE denoising enhancement module in series. ABlock is a region attention mechanism natively introduced in YOLOv13. Its core idea is to divide the feature map into several groups along the channel dimension, with each group independently performing self-attention computation within a local window, thereby achieving global receptive field modeling at extremely low computational cost. ABlock effectively captures long-range dependencies and contextual information of defect targets, but lacks dedicated suppression mechanisms for high-frequency noise, such as rolling textures and reflections that widely exist in industrial images. To address this, this paper cascades the DE module after ABlock, forming a collaborative processing chain of “attention focusing—noise suppression—texture enhancement”: ABlock first completes attention focusing and contextual modeling of defect regions, and then the DE module performs denoising and edge texture enhancement on the attention-enhanced features, preserving the global modeling advantages of ABlock while compensating for its insufficient anti-interference capability in industrial scenarios. The outputs of each cascaded module are retained and channel-concatenated with the initial feature map $x_{\mathrm{msf}\_\mathrm{out}}$, as shown in Equation (3). The concatenated feature map is then transformed by a 1×1 convolution (cv2) to match the output channel dimension, generating the feature map $x_{\mathrm{out}}$. Finally, a learnable scaling coefficient γ is used to perform residual fusion between the input feature map x and $x_{\mathrm{out}}$, as shown in Equation (4), which effectively preserves the integrity of gradient flow and alleviates the vanishing gradient problem in deep networks.

(3) $$y=\left[x_{{\mathrm{msf}}_{\mathrm{out}}}\right]\cup \{m(y[-1])|m\in M\}$$

(4) $$x_{\mathrm{final}}=x+\gamma \cdot {\mathrm{Conv}}_{1\times 1}({\mathrm{Concat}}_{\mathrm{channel}}\left(y)\right)$$

where M is a list of n cascaded ABlock-DE modules, y[−1] denotes the output of the previous module, and ∪ denotes the concatenation operation along the channel dimension.

#### 3.2.1. Adaptive Weight Multi-Scale Fusion Branch MSKF

The MSKF multi-scale multi-kernel fusion branch module adopts a parallel branch convolution structure, as shown in Figure 1b, to adapt to defect features of different scales. The 1×1 convolution (cv1) branch preserves the original detail information of the input features, focusing on capturing texture features of small-scale defects such as pixel-level elongated cracks and tiny pitting, avoiding the destruction of weak features by large convolution kernels. The 3×3 convolution (cv2) branch expands the receptive field, focusing on capturing global semantic features of large-scale defects such as patches and inclusions. The design of grouped convolution simultaneously reduces the computational cost of convolution, addressing the issues of incomplete large-defect feature extraction and high computational cost in the original module. To achieve adaptive fusion of features from different branches, the module introduces a learnable weight parameter ω, normalizes the weights via the Softmax function, enabling the model to automatically learn the optimal fusion weights for defects of different scales during training. The fused features are then fed into the SEL lightweight channel attention module to perform channel-wise feature enhancement. The computational process is shown in Equations (5)–(7). This design allows the module to dynamically adjust the fusion strategy according to the scale characteristics of the input defects: when the input is dominated by tiny defects, the 1×1 branch receives higher weight; when the input is dominated by large-scale defects, the weight of the 3×3 branch increases accordingly, thereby achieving adaptive balance of multi-scale features.

(5) ω=Softmax(ω)

(6) $$x_{\mathrm{fused}}=\omega_{0}\cdot {\mathrm{Conv}}_{1\times 1}\left(x\right)+\omega_{1}\cdot {\mathrm{DSConv}}_{3\times 3}\left(x\right)$$

(7) $$\mathrm{MSF}\left(x\right)=\mathrm{SEL}(x_{\mathrm{fused}})$$

where $\omega \;\in {\;R}^{2}$ is a learnable weight parameter, and $\omega_{0}$ and $\omega_{1}$ are the normalized adaptive weights.

#### 3.2.2. Lightweight Channel Attention Mechanism SEL

For the SEL lightweight channel attention module, global channel information is first obtained via adaptive average pooling, followed by two 1×1 convolutions, LayerNorm, SiLU activation, and a Sigmoid activation function to generate channel attention weights, as shown in Figure 1d. The complete computational flow of the module is presented in Equations (8)–(10). The SEL module incorporates two lightweight and stability optimization strategies in its design. First, it uses LayerNorm instead of BatchNorm, and the dimension on which LayerNorm operates is precisely specified in the code to match the feature dimension after global average pooling. This design addresses the issue of BatchNorm statistic shifts during small-batch training in industrial defect detection scenarios, because industrial datasets are often limited in size and distributed training strategies may lead to unstable estimation of BatchNorm mean and variance. The instance-level normalization property of LayerNorm effectively improves the stability of model training. Second, the bias parameters of the two 1×1 convolutional layers are removed (bias = False), which reduces the number of model parameters while avoiding interference from bias terms in channel weight learning, thereby enhancing the accuracy of attention weights.

(8) $$x_{\mathrm{avg}}=\mathrm{AvgPool}(x)$$

(9) $$\mathrm{att}=\mathrm{Sigmoid}({\mathrm{Conv}}_{1\times 1}(\mathrm{SiLU}(\mathrm{LayerNorm}({\mathrm{Conv}}_{1\times 1}(x_{\mathrm{avg}})))))$$

(10) $$\mathrm{SEL}\left(x\right)=x\cdot \mathrm{att}$$

#### 3.2.3. Denoising and Texture Enhancement Module DE

To address the poor robustness of the original module against background noise on steel surfaces, this paper introduces a denoising and texture enhancement module (DE) after the ABlock module, which simultaneously suppresses background noise and enhances defect edge features following attention-based feature extraction. The design rationale for placing the DE module after ABlock is as follows: ABlock performs initial localization of defect regions and contextual modeling via the region attention mechanism, but the attention output features may still retain some background noise, and the fine texture of defect edges has not yet been strengthened. The DE module is specifically designed for post-processing these issues, forming a complete processing chain of “localization—denoising—enhancement”, so that features achieve a better signal-to-noise ratio and edge clarity before entering the next network layer.

The module adopts lightweight depthwise separable convolution (DSConv) as its core and performs feature optimization in two stages, as shown in Figure 1c. The first stage is denoising, where a 3×3 depthwise separable convolution performs spatial convolution on each channel independently, removing random noise such as rolling textures and reflections from steel surfaces while preserving the spatial location information of defects to the greatest extent. Compared with standard convolution, depthwise separable convolution decouples inter-channel fusion from spatial feature extraction, reducing the number of parameters and computational cost, while avoiding excessive smoothing of weak defect features caused by standard convolution. The second stage is texture enhancement, where a 1×1 pointwise convolution performs inter-channel fusion on the denoised features, strengthening the edge texture features of elongated cracks and improving the feature discriminability between defects and backgrounds, thus solving the problem of blurred defect edge features in the original module. The core computational process of the module is shown in Equation (11).

(11) $$\mathrm{DE}(x)={\mathrm{Conv}}_{1\times 1}({\mathrm{DSConv}}_{3\times 3}\left(x\right))$$

#### 3.2.4. Dual Learnable Residual Scaling Structure

To ensure gradient flow integrity and feature reuse capability during deep network training, two learnable residual scaling coefficients are introduced into the A2C2f-MSDE module, located at different positions within the module and undertaking distinct optimization functions.

The first scaling coefficient $\gamma_{\mathrm{msf}}$ is embedded in the residual connection of the MSKF branch, as shown in Equation (2). This coefficient controls the fusion ratio between the multi-scale fused features and the original features, allowing the network to adaptively balance the weights between “preserving original features” and “incorporating multi-scale enhanced features” during training. The initial value is set to a small number, so that the original features dominate at the early training stage, and the contribution of multi-scale features is gradually increased as training proceeds, avoiding the adverse impact of early random perturbations on feature learning.

The second scaling coefficient γ is embedded in the global residual connection of the module, as shown in Equation (4). This coefficient controls the fusion ratio between the module output and the module input, forming a cross-module gradient highway. In deep networks, gradients need to back-propagate through multiple modules. If each module adopts an identity mapping residual connection, gradient attenuation will be significantly aggravated. By introducing a learnable γ, the network can dynamically adjust the contribution of the residual path during training, ensuring that gradients can be efficiently transmitted back to shallow layers and effectively alleviating the vanishing gradient problem.

### 3.3. Module Synergy Mechanism and Comparative Analysis

The proposed A2C2f-MSDE module possesses four core innovations. First, the MSKF multi-scale multi-kernel fusion branch with learnable adaptive weights dynamically balances the capture of local detail features of tiny defects and global semantic features of large-area defects, strengthening the multi-scale feature representation capability for steel surface defects. Second, the embedded lightweight SEL channel attention module achieves adaptive weighting of channel features, enhancing the response of effective defect features while suppressing the weights of background noise channels, effectively improving the signal-to-noise ratio of defect features. Third, the dedicated DE module further purifies defect features after the attention module, enhancing the edge and texture features of low-contrast defects, and significantly improving the model’s discriminative ability for irregular, low-contrast defects. Fourth, two learnable residual scaling coefficients are embedded in the multi-scale fusion branch and the main residual path, effectively preserving the integrity of gradient flow in deep networks, alleviating the vanishing gradient problem, and improving the training convergence stability and feature reuse capability of the model.

Compared with the original A2C2f module in terms of structure and function, this module innovatively achieves three major improvements based on the original A2C2f architecture: multi-scale dynamic feature fusion, dual-branch feature enhancement and denoising, and dual learnable residual optimization. This optimized design addresses the limitations of the original A2C2f module in steel surface defect detection, namely insufficient feature extraction for tiny defects, weak anti-interference capability, and training instability. While preserving gradient propagation integrity, it maximizes the extraction of discriminative features of multi-scale steel defects. The A2C2f-MSDE module achieves superior comprehensive feature extraction performance compared to the original module.

## 4. Experiment and Analysis

### 4.1. Experimental Setup Details

We conducted a series of performance experiments to comprehensively validate the proposed YOLOv13-A2C2f-MSDE steel surface defect detection model. This chapter systematically analyzes the detection accuracy, lightweight characteristics, and industrial scenario adaptability of the improved model from the aspects of experimental environment and datasets, model evaluation metrics, experimental hyperparameter settings, ablation studies, comparative experiments, and generalization tests.

#### 4.1.1. Experimental Environment

The computing platform is the fundamental prerequisite for rigorous verification of architectural optimization effects. The hardware and software environment configurations in this experiment are kept fixed to ensure the uniqueness of variables across all comparative and ablation experiments. This paper adopts a graphics workstation as the operating platform for all model frameworks. The specific configuration is as follows: CPU is Intel Xeon E5-2698v3 (Intel Corporation, Santa Clara, CA, USA), GPU is NVIDIA Tesla V100-SXM2-32GB (NVIDIA Corporation, Santa Clara, CA, USA), and RAM is 64GB. The software configuration includes CUDA 12.1 and cuDNN 8.9.0 (NVIDIA Corporation, Santa Clara, CA, USA), and the operating system is Linux Ubuntu 20.04 LTS (Canonical Ltd., London, UK). The compilation environment is built on Python 3.11.14 and the deep learning framework PyTorch 2.2.2 (Meta AI, Menlo Park, CA, USA). To ensure fairness and reliability in performance comparison experiments, all models are initialized before each training session, and the relevant hyperparameters are strictly kept consistent.

#### 4.1.2. Experimental Datasets

**Figure 2:**
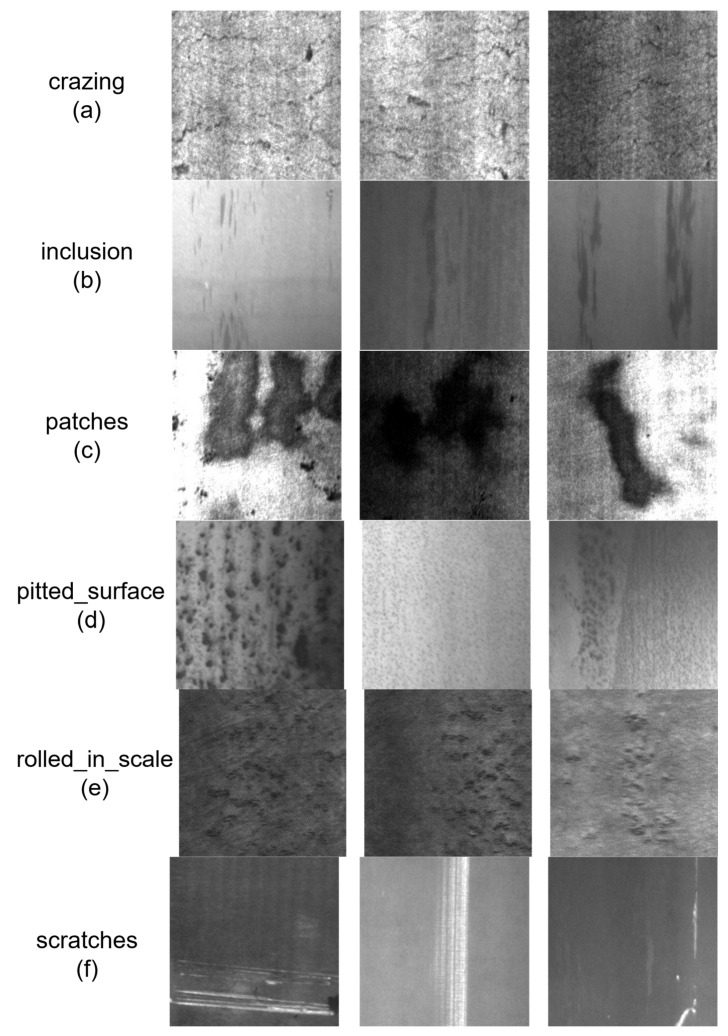
Examples of the six defect types in the NEU-DET dataset used for model training.

The experiments in this paper adopt the public NEU-DET strip steel surface defect dataset released by Northeastern University, Shenyang, China. This dataset is a classic benchmark in the field of industrial metal surface defect detection, covering the six most common types of surface defects in the hot-rolled strip steel production process, including cracks, inclusions, patches, pitted surfaces, rolled-in scale, and scratches, as shown in Figure 2, which meets the practical requirements of industrial strip steel quality inspection. The dataset contains a total of 1800 grayscale images, with 300 images per defect type, and the image resolution is uniformly 200 × 200 pixels. This paper divides the dataset into training, validation, and test sets in an 8:1:1 ratio, with 1440 images for training, 180 for validation, and 180 for testing.

**Figure 3:**
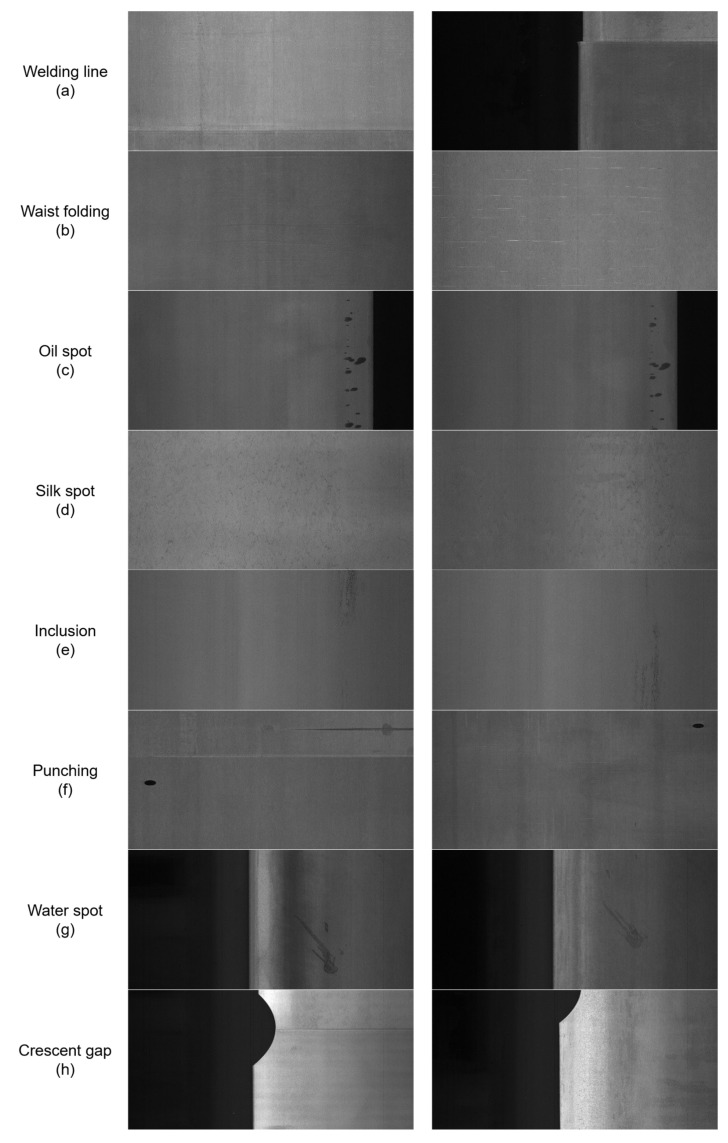

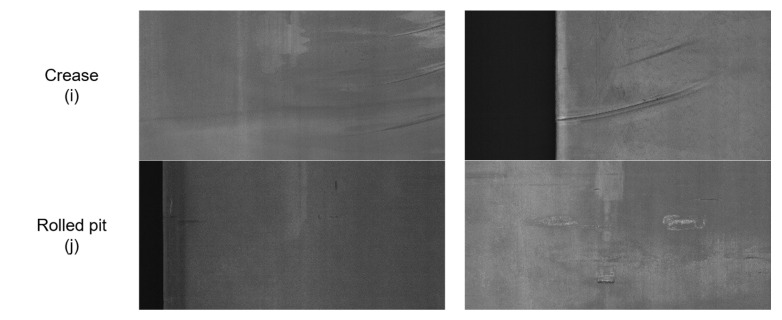
Examples of the ten defect types in the GC10-DET dataset used for model generalization testing.

The generalization test in this paper adopts the public GC10-DET steel surface defect dataset, released by the Institute of Automation, Chinese Academy of Sciences, Beijing, China. This dataset consists of surface defect data collected during the steel plate manufacturing process, captured by grayscale cameras with an image resolution of 2048 × 1000. It contains a total of 10 types of surface defects, including welding line, waist folding, oil spot, silk spot, inclusion, punching, water spot, crescent gap, crease, and rolled pit, as shown in Figure 3. After removing invalid data, the dataset contains a total of 2292 images, which are divided into training, validation, and test sets in the same 8:1:1 ratio as the previous experiments.

### 4.2. Model Evaluation Metrics and Experimental Hyperparameter Settings

Comprehensive and objective model evaluation metrics serve as the core basis for quantifying model performance and guiding iterative model optimization. The confusion matrix, as a fundamental tool for classification model evaluation, intuitively presents the correspondence between model predictions and ground-truth labels. Through statistical analysis of the four core quantities of classification results, it can systematically dissect the classification performance of a model [19]. For the task of strip steel surface defect detection, this study builds upon the core accuracy metrics commonly used in object detection, while also incorporating evaluation indicators related to model lightweight and inference speed in response to the real-time detection and edge deployment requirements of industrial settings, thereby constructing a complete model evaluation system covering accuracy, efficiency, and deployment feasibility.

This paper adopts Precision (P), Recall (R), and mean Average Precision (mAP) as detection accuracy metrics. Precision represents the proportion of correctly detected defect targets among all detected targets in the model’s predictions, reflecting the model’s ability to resist false positives. Recall represents the proportion of correctly detected defect targets among all ground-truth defect targets in the dataset, reflecting the model’s ability to resist false negatives. This paper employs three mean Average Precision metrics, namely mAP50, mAP75, and mAP50-95. Before elaborating on these three metrics, the concept of Intersection over Union (IoU) should first be clarified. IoU measures the overlap between the predicted bounding box and the ground-truth bounding box, with a value in (0,1); a higher value indicates a higher degree of overlap between the predicted box and the ground-truth box. mAP50 is the mAP value when the IoU threshold is set to 0.5, used to evaluate the detection rate of the model. mAP75 is the mAP value when the IoU threshold is set to 0.75, comprehensively emphasizing localization accuracy and bounding box quality. mAP50-95 is the average mAP over all IoU thresholds from 0.5 to 0.95 with a step size of 0.05, used to evaluate the overall detection quality of the model, including bounding box localization accuracy and classification accuracy.

Considering the real-time requirements and edge deployment demands of industrial strip steel quality inspection, this paper introduces three lightweight-related metrics: the number of parameters (Parameters), the amount of computation (Giga Floating-point Operations, GFLOPs), and the frame rate (Frames Per Second, FPS). The number of parameters refers to the total number of trainable parameters in the model, which directly determines the model’s storage footprint and hardware requirements for deployment. The amount of computation is the number of floating-point operations required for a single forward inference, characterizing the model’s computational complexity. FPS indicates the number of image frames processed per second, serving as a metric for evaluating image processing or model inference speed.

All experiments in this paper are carried out on YOLOv13 under the Ultralytics framework (Ultralytics, Los Angeles, CA, USA). During training, hyperparameters are kept largely consistent, with the batch size adjusted according to model size. Only the backbone network and feature fusion module structures are modified, ensuring the comparability of experimental results. The core training hyperparameters are shown in Table 1.

### 4.3. Ablation Experiments of the A2C2f-MSDE Module

**Figure 4:**
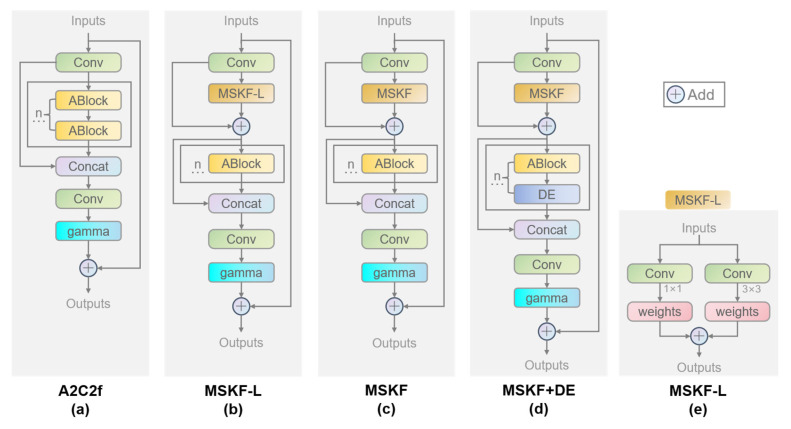
Architecture diagrams of models in the ablation experiments: (**a**) architecture of the original A2C2f module, n represents that there are n ABlock modules, and the number n can be specified in the model; (**b**) architecture after introducing MSKF-L based on (**a**); (**c**) architecture after introducing SEL based on (**b**); (**d**) architecture after introducing the DE module based on (**c**), n represents that there are n combinations of ABlock modules and DE modules, and the number n can be specified in the model; (**e**) architecture of MSKF-L.

**Figure 5:**
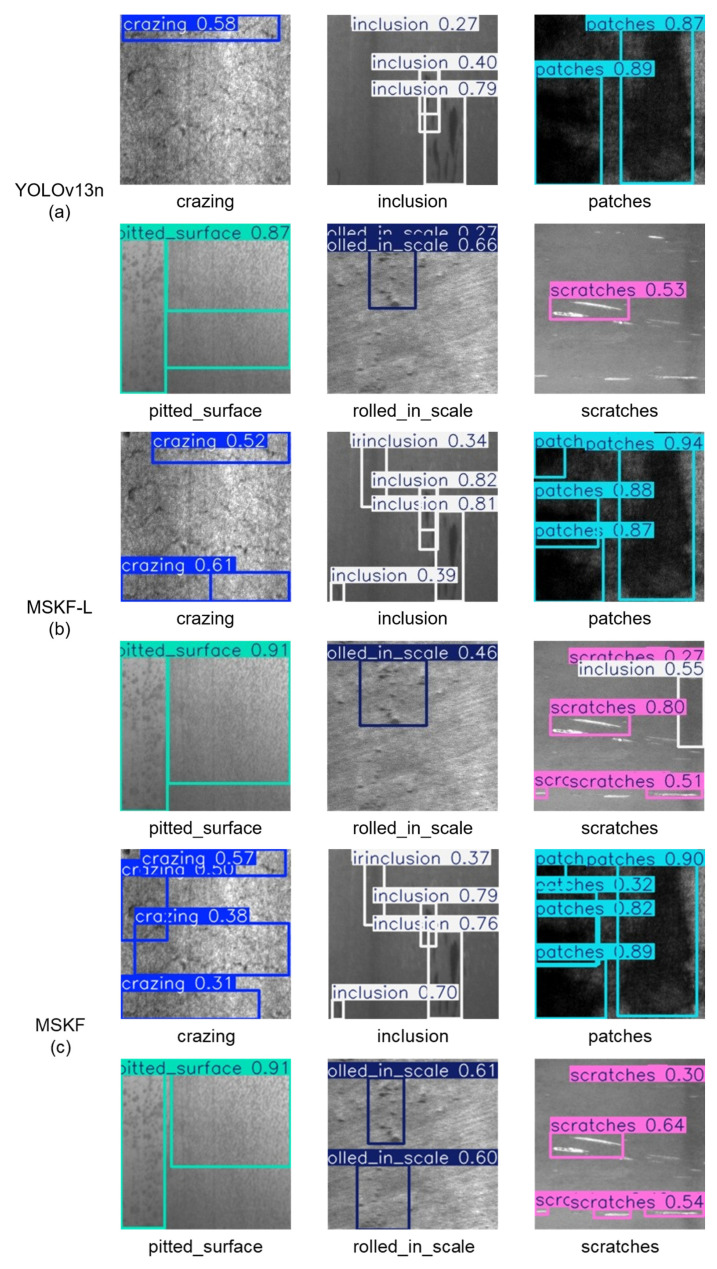

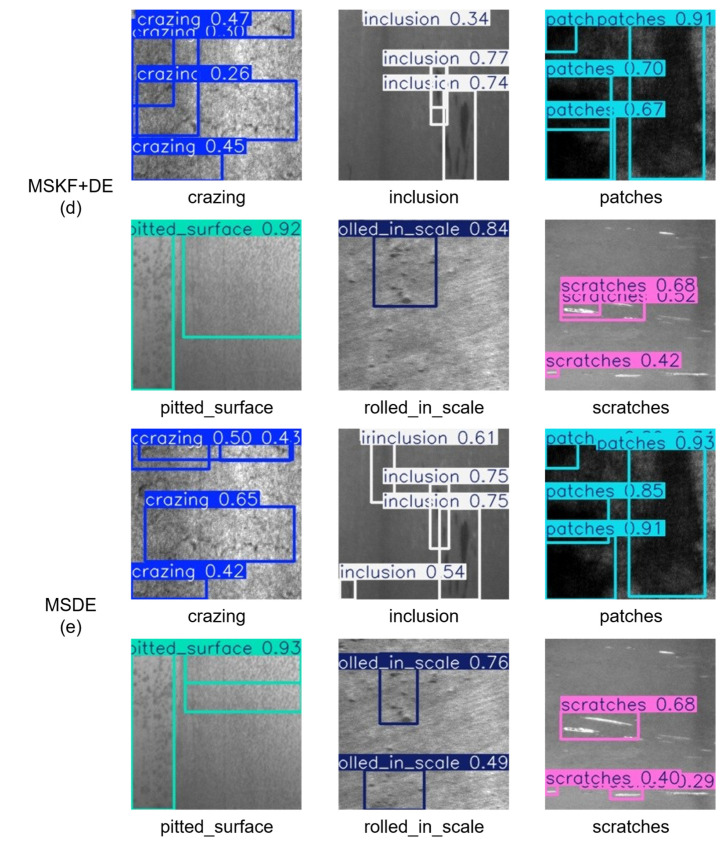
Visualization of inference results of each model in the ablation experiments on the NEU-DET strip steel surface defect dataset. (**a**) shows the detection results of the YOLOv13n model on various defects; (**b**) shows the detection results after incorporating the MSKF-L module; (**c**) shows the detection results after incorporating the MSKF module; (**d**) shows the detection results after incorporating both the MSKF and DE modules; (**e**) shows the detection results after incorporating the MSDE module.

The purpose of the ablation experiments is to verify the performance gains of the proposed A2C2f-MSDE module over the YOLOv13 baseline model and to clarify the effectiveness and contribution of the improved module. Our ablation experiments take the native YOLOv13-n as the baseline model and adopt the single-variable control method to validate, one by one, the independent performance contributions and synergistic gains of the four core subcomponents of the proposed A2C2f-MSDE module: the MSKF multi-scale fusion branch, the SEL lightweight channel attention module, the DE denoising and texture enhancement module, and the optimized residual structure with dual learnable scaling coefficients. The adaptation value of each improvement for the strip steel surface defect detection task is determined. The model structures used in the ablation experiments are shown in Figure 4, and the visual comparison of experimental results is shown in Figure 5. The changes in model accuracy, parameter count, and computational complexity before and after the improvements are compared. The experimental design and results are presented in Table 2.

When the MSKF-L multi-scale fusion branch was introduced, the module structure is shown in Figure 4b. As can be seen in Table 2, the model parameter count decreased to 2.248 M, an 8.2% reduction from the baseline; GFLOPs decreased to 6.0, a 3.2% reduction; mAP75 significantly increased from 0.391 to 0.443, a gain of 5.2 percentage points; and mAP50-95 increased to 0.431, a gain of 1.6 percentage points. These results verify that the MSKF-L branch, while reducing computational cost, captures both pixel-level texture details of tiny defects and global semantic features of large-scale defects. It enhances the dynamic balance of multi-scale features and significantly improves the model’s localization accuracy for defect bounding boxes, leading to a substantial gain in the mAP75 metric at a high IoU threshold. As shown in Figure 5b, the localization accuracy for both large-scale and tiny defects is significantly improved, the overlap between predicted boxes and ground-truth defect regions is greatly increased, and the miss rate is markedly reduced. For example, the detection rates of large-scale defects such as patches and scratches, as well as tiny defects such as crazing and inclusion, are clearly improved. The prediction confidences for inclusion and scratches increased significantly by 0.3–0.4 (inclusion: 0.40 → 0.82, scratches: 0.53 → 0.80), validating the collaborative multi-scale defect feature capture capability of the MSKF-L branch.

After adding the SEL lightweight channel attention module on top of the MSKF-L branch, the complete MSKF module is formed, with its structure shown in Figure 4c. As can be seen in Table 2, the model’s mAP50 jumped from 0.744 to 0.764, a gain of 2.0 percentage points; mAP50-95 also increased to 0.433, while the parameter count and GFLOPs increased only negligibly, preserving the lightweight characteristics. In the experiment where only the SEL module was removed from the complete module, all three mAP metrics decreased: mAP50 dropped from 0.744 to 0.756, mAP75 dropped from 0.466 to 0.416, and mAP50-95 dropped from 0.454 to 0.438. These results verify the effectiveness of the SEL module. From the visual results in Figure 5c, it can be seen that the responses of effective defect features are further enhanced, the prediction confidences for various defect types are generally improved, and background false positives are significantly reduced. For example, in the experiment without the SEL module, scratch defects were misclassified as inclusions. The detection rate of low-contrast defects is notably enhanced, such as a new detection box with confidence 0.38 for crazing and a new detection box with confidence 0.60 for rolled-in scale. These results demonstrate that the SEL module, without increasing computational burden, achieves adaptive weighting of channel features, strengthens the feature responses of defect-related effective channels, improves the signal-to-noise ratio of defect features, enhances the model’s ability to recognize low-contrast weak defects, and reduces both false positives and false negatives, thereby achieving a significant improvement in the mAP50 metric, which characterizes detection rate.

After introducing the DE denoising and texture enhancement module on top of MSKF, the module structure is shown in Figure 4d. As can be seen in Table 2, the model’s mAP75 increased to 0.443, and mAP50-95 further increased to 0.438, with only a very small increase in parameter count and GFLOPs. Similarly, in the experiment where only the DE module was removed from the complete module, all three mAP metrics decreased, verifying the effectiveness of the DE module. From the visual results in Figure 5d, it can be seen that the edge features of low-contrast defects under complex rolling backgrounds are effectively enhanced, and the discriminability between defects and backgrounds is improved. For example, the prediction confidence for rolled-in scale increased by 0.23 (rolled-in scale: 0.61 → 0.84). These results confirm that the DE module, while removing random noise from the strip steel surface, preserves the spatial location information of defects to the greatest extent, strengthens the edge texture features of tiny defects such as crazing and pitted surface, and further consolidates the model’s localization accuracy and comprehensive detection performance.

Based on MSKF + DE, after introducing the optimized residual structure with dual learnable scaling coefficients, the complete A2C2f-MSDE module is formed, with its structure shown in Figure 1a. As can be seen in Table 2, the model achieves a simultaneous significant leap in all accuracy metrics. Compared with the original YOLOv13n model, mAP50 increased from 0.742 to 0.774, mAP75 increased from 0.391 to 0.466, and mAP50-95 increased from 0.415 to 0.454, while the parameter count decreased by 4.6% and GFLOPs also decreased correspondingly, imposing no additional computational burden; moreover, the FPS remained above 500, which is a considerable figure that further ensures the model’s lightweight nature. From the visual results in Figure 5e, it can be seen that the detection rates and prediction confidences for various defect types have increased substantially compared with the original YOLOv13n model, while the gaps between prediction confidences for different defect types have decreased, effectively balancing detection rate and prediction confidence. These results fully demonstrate that the optimized residual structure, through the design of dual learnable scaling coefficients, effectively preserves the integrity of gradient flow in deep networks, alleviates the vanishing gradient problem, greatly improves the training convergence stability of the model, and fully unleashes the optimization effects of the aforementioned multi-scale fusion, channel attention, and denoising enhancement modules. It serves as the core foundation for achieving synergistic gains among the subcomponents.

### 4.4. Comparison with Mainstream Object Detection Models

To verify the performance advantages of the proposed A2C2f-MSDE model, this paper uses YOLOv13n as the baseline platform and conducts benchmark comparisons with object detection models published in recent years at top computer vision conferences such as CVPR and CVF. All comparison models are trained and validated under exactly the same dataset, experimental environment, and training parameters to ensure fairness of the experimental results.

**Figure 6:**
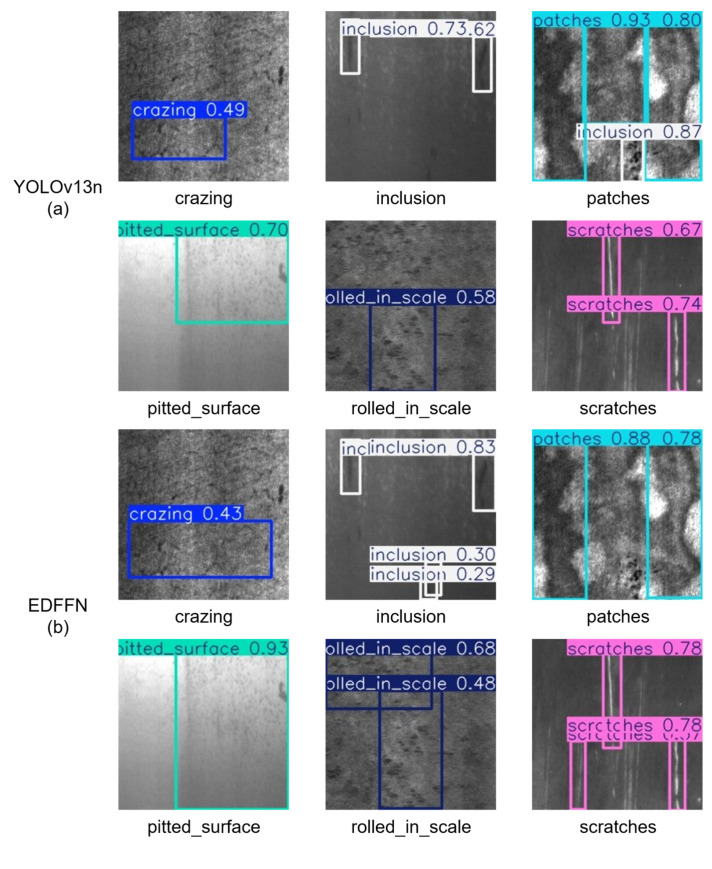

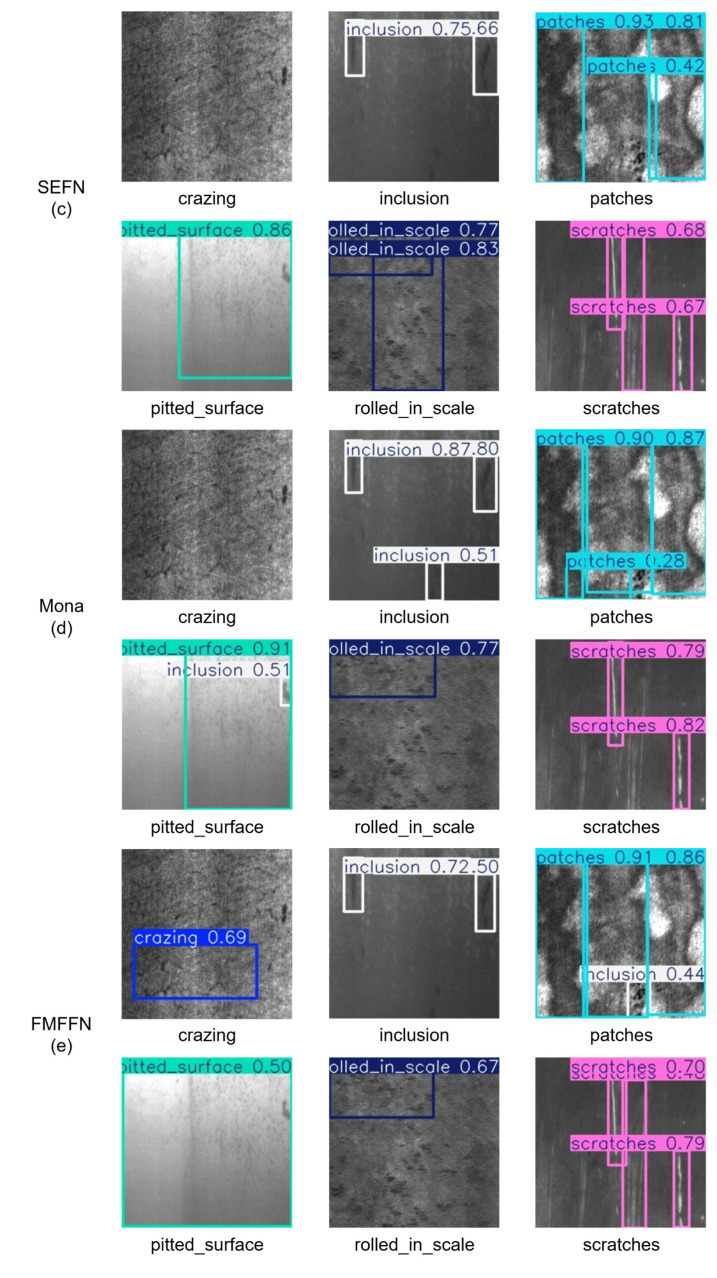

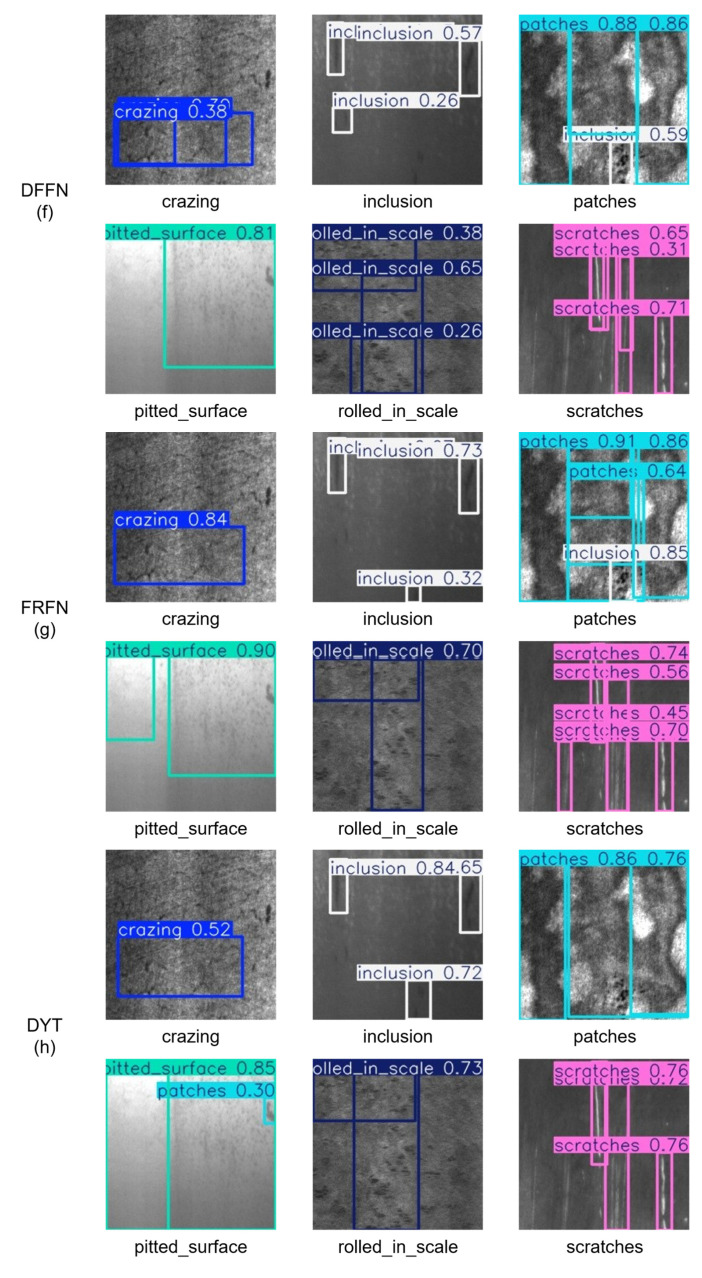

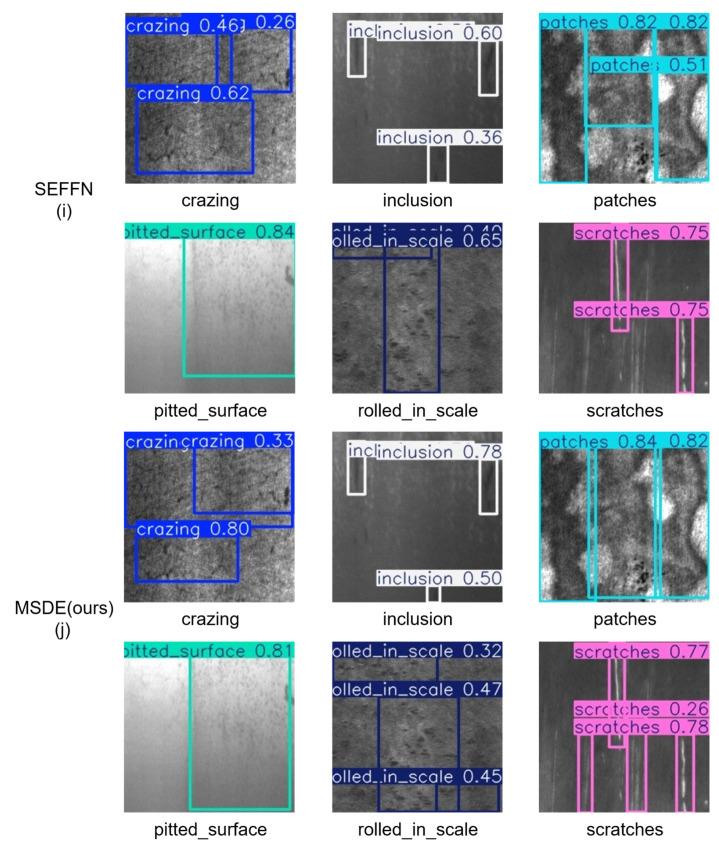
Visualization of inference results of each model in the comparative experiments on the NEU-DET strip steel surface defect dataset.

The test results in Table 3 show that our model achieves comprehensive improvements in detection accuracy. Its comprehensive evaluation metric mAP50-95, which characterizes overall detection performance, reaches 0.454, an absolute increase of 0.039 over the native YOLOv13n baseline model, representing a relative gain of 9.4%. Compared with the FMFFN improved model, which has the second-best overall performance in the comparative experiments, our model still achieves an improvement of 0.018, a relative gain of 4.1%, while outperforming mainstream lightweight models such as EDFFN, Mona, and DynamicTanh by more than 0.020. As shown in the visual comparison in Figure 6, the YOLOv13n baseline model has significant performance shortcomings, with severe missed detection issues for various defect types, and a large number of subtle defects are not effectively detected. Although mainstream comparison models such as EDFFN, FMFFN, DFFN, and FRFN show some performance improvements over the baseline, their improvements in addressing the issues of repeated detection, false positives, and missed detections for tiny defects are limited. The missed detection problem is particularly prominent in the SEFN and Mona models, while FMFFN, DFFN, and FRFN introduce a large number of background false positives to improve recall. The proposed MSDE module significantly outperforms all comparison models in terms of detection rate and overall prediction confidence across various defect types. For tiny defects such as crazing and rolled-in scale, it achieves the highest detection rate, with prediction confidence increased by more than 0.3 over the baseline (crazing: 0.49 → 0.80, rolled-in scale: 0 → 0.32). For large-scale defects such as patches, the predicted bounding boxes closely align with the actual defect regions, with no repeated detection or category misclassification issues. For low-contrast defects such as inclusions and scratches, it achieves relatively continuous and complete detection (inclusions: 0 → 0.50, scratches: 0 → 0.78), with stable confidence distribution and no significant fluctuation. These results fully validate that the A2C2f-MSDE module, through the synergistic design of multi-scale feature fusion, lightweight channel attention, denoising enhancement mechanisms, and optimized residual structure, can generate higher-quality feature representations of steel defects. The mAP50 metric, which characterizes the model’s defect detection rate, reaches 0.774, outperforming all comparison models and achieving the best detection effect, an improvement of 0.032 over the baseline model. This fully demonstrates the model’s comprehensive detection capability for steel surface defects of various morphologies and scales, effectively reducing the risk of missed detections in industrial scenarios. The mAP75 metric, which characterizes detection performance under high localization accuracy requirements, reaches 0.466, a substantial increase of 0.075 over the baseline model, representing a relative gain of 19.2%, and an improvement of 0.019 over the second-best DynamicTanh model, a relative gain of 4.3%. This significant improvement in mAP75 confirms the edge texture enhancement effect of the DE denoising enhancement module and the contour feature capture capability of the MSKF multi-scale fusion branch, improving boundary localization for low-contrast and irregular defects. Furthermore, in terms of balanced optimization of classification discrimination performance, the model achieves a precision of 0.805, matching the best Mona model in the comparison group, while achieving a recall of 0.713, which is 0.045 higher than the Mona model, achieving an optimal balance between precision and recall. It is worth noting that the FRFN and FMFFN models, which have the highest recall in the comparison group, both trade precision for recall gains: the FRFN model achieves the highest recall of 0.742 in the group but only 0.715 precision, which is 0.09 lower than our model; the FMFFN model achieves a recall of 0.729 but only 0.750 precision, which is 0.055 lower than our model. In steel industrial quality inspection scenarios, excessively high false positive rates significantly increase the production cost of manual re-inspection, while excessively low recall rates can lead to non-conforming products entering the market, causing serious safety accidents. Our model achieves the highest precision in the comparison group while also achieving a leading recall, demonstrating that the model reduces both false positives and missed detections in industrial quality inspection, meeting the requirements of industrial on-site quality inspection. It is worth noting that although Mona achieves a precision of 0.805, its mAP50-95 is only 0.433, which is significantly lower than our model.

Computational efficiency analysis shows that the GFLOPs of our model are only 6.1, a slight decrease from 6.2 of the native YOLOv13n, indicating that no additional computational burden is incurred despite a substantial improvement in accuracy. Compared with lightweight advanced models such as FMFFN and DynamicTanh, our model achieves a leap in detection accuracy with only a negligible increase in computational cost. Specifically, compared with the Spatially-Enhanced Feedforward Network model, our model reduces computational cost by 18.7% while improving mAP50-95 by 0.029. Compared with the Mona model, it reduces computational cost by 10.3% while improving mAP50-95 by 0.021. Compared with the EDFFN model, it reduces computational cost by 4.7% while improving mAP50-95 by 0.022. These results demonstrate that our model achieves rational allocation of computational resources rather than trading performance for unrestrained increases in computational cost.

### 4.5. Generalization Test

To comprehensively evaluate the cross-dataset generalization capability of the proposed A2C2f-MSDE module and verify its robustness and adaptability in industrial scenarios different from the training data distribution, this paper conducts a generalization test on the public GC10-DET steel surface defect dataset. The GC10-DET dataset is collected during the steel plate manufacturing process, with an image resolution of 2048 × 1000, containing 10 different types of surface defects. Its defect morphologies and imaging conditions differ significantly from those of the NEU-DET dataset, which effectively tests the generalization performance of the model. During the test, the model parameters remain exactly the same as those in the NEU-DET experiment, without any fine-tuning or parameter adaptation for the GC10-DET dataset, ensuring the objectivity and comparability of the generalization evaluation.

The generalization test results in Table 4 show that the proposed A2C2f-MSDE model maintains stable detection performance on the GC10-DET dataset. Compared with the native YOLOv13n baseline model, the A2C2f-MSDE model improves mAP50-95 from 0.369 to 0.388, an absolute gain of 0.019 (relative gain of 5.1%); mAP75 increases from 0.340 to 0.373, an absolute gain of 0.033 (relative gain of 9.7%); and recall increases from 0.614 to 0.672, an absolute gain of 0.058 (relative gain of 9.4%). The consistent improvement of the above metrics fully verifies that the design effectiveness of the A2C2f-MSDE module in multi-scale feature fusion, noise suppression, and edge texture enhancement remains stable across different industrial scenarios, demonstrating good cross-dataset generalization capability.

**Figure 7:**
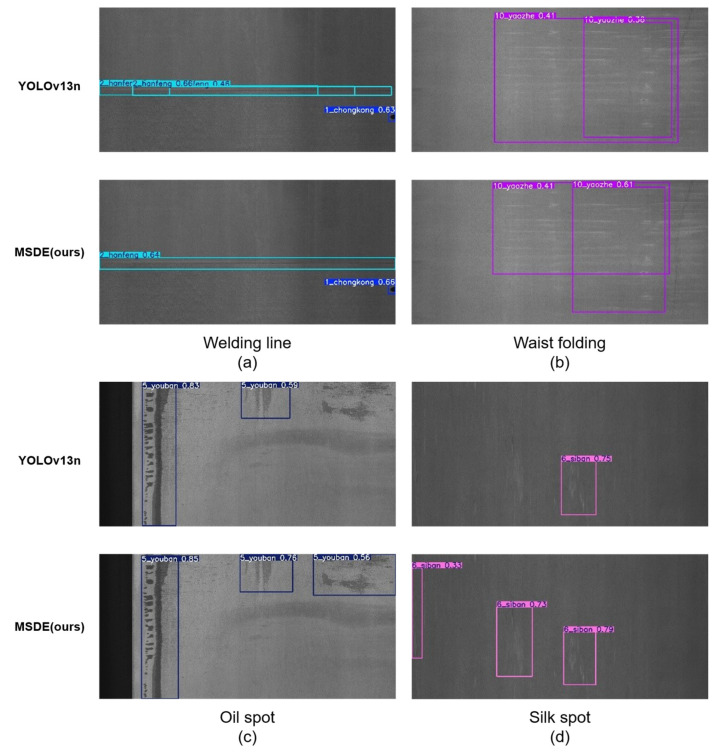

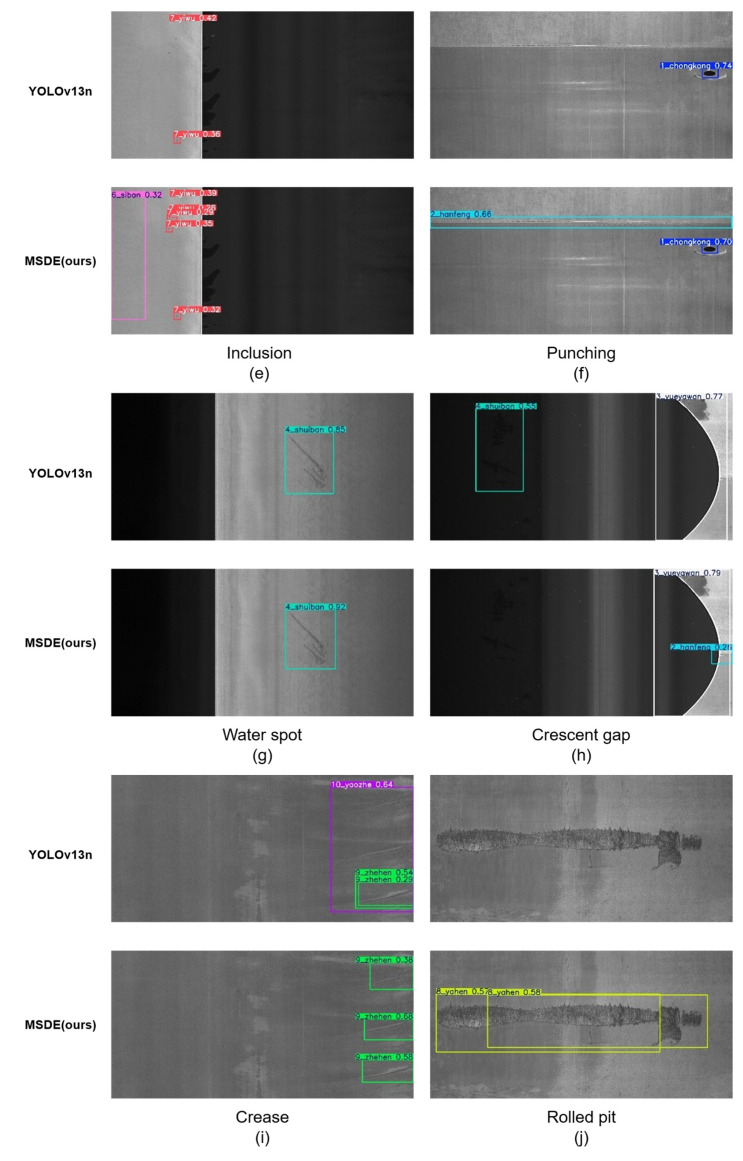
Visualization of inference results of each model in the generalization test experiment on the GC10-DET steel surface defect dataset.

From the visualization results in Figure 7, it can be seen that for long, continuous defects such as welding lines in the complex scenarios of the GC10-DET dataset, the native YOLOv13n baseline model suffers from severe fragmented detection and incomplete bounding box localization, only covering local regions of the defects. For scattered tiny defects such as oil spots, silk spots, and inclusions, the baseline model exhibits prominent missed detection issues, with a large number of low-contrast, small-pixel-ratio defects not effectively detected, failing to meet the detection requirements for tiny defects in high-resolution images. In stark contrast, the proposed A2C2f-MSDE model achieves stable performance improvements across all ten types of typical steel surface defects in the GC10-DET dataset, with reduced miss rates and improved localization accuracy. In terms of detection completeness, the model improves the detection rate of scattered tiny defects such as silk spot and inclusion; weak edge features of crescent gaps that were missed by the baseline model are successfully detected; long continuous defects such as welding line and crease are detected continuously and completely, with reduced fragmentation, missed detections, and false positives, directly corresponding to the 9.4% relative gain in recall. In terms of defect localization accuracy, the model significantly improves the alignment between predicted bounding boxes and ground-truth defect contours for various defect types; bounding boxes for large-scale defects such as waist folding and rolled pit completely cover the full length and entire region of the defects, reducing issues of localization offset or insufficient coverage, fully confirming the 9.7% relative improvement in mAP75 and validating that the module can stably achieve defect edge texture enhancement and precise localization across different scenarios.

It is worth noting that on the GC10-DET dataset, the mAP50 of the MSDE model is 0.704, which is essentially comparable to the baseline model’s 0.702, indicating that the model maintains stable overall defect detection capability. However, the precision metric decreases from 0.799 to 0.710, showing a certain degree of decline. This phenomenon can be attributed to the distribution differences between the GC10-DET dataset and the NEU-DET dataset: the GC10-DET dataset contains 10 defect categories, more than NEU-DET, and some defect categories (e.g., waist folding, silk spot) have extremely low discriminability from background textures. Additionally, the test images have higher resolution and more complex background regions, leading the model to introduce more false positives while maintaining high recall. This phenomenon also indirectly confirms that, without any adaptation to the new dataset, the model achieves significant improvements in recall and localization accuracy at the cost of an acceptable drop in precision, demonstrating strong cross-scenario transfer capability.

In summary, the proposed A2C2f-MSDE module maintains overall defect detection capability while significantly improving detection performance and recall under high localization accuracy requirements, validating its generalization capability and robustness across different industrial scenarios.

## 5. Conclusions

To address the core problems of the native A2C2f module in YOLOv13 for steel surface defect detection—namely, insufficient multi-scale feature extraction for tiny defects, weak anti-interference capability under complex industrial backgrounds, and vanishing gradient and training instability in deep networks—this paper proposes a multi-scale denoising enhanced module, A2C2f-MSDE.

Regarding Objective 1, the MSKF multi-scale multi-kernel fusion branch with learnable adaptive weights achieves a dynamic balance between local details of tiny defects and global semantics of large-scale defects, with mAP75 increasing from 0.391 to 0.466 (a relative gain of 19.2%). Regarding Objective 2, the lightweight SEL channel attention module and DE denoising enhancement module work synergistically to suppress background noise and strengthen edge textures, raising mAP50 from 0.742 to 0.774 and significantly improving detection confidence for low-contrast defects such as rolled-in scale (0.61 → 0.84). Regarding Objective 3, the dual learnable residual scaling structure preserves gradient flow integrity and stabilizes training, enabling all accuracy metrics to rise simultaneously while reducing the parameter count by 4.6%. Regarding Objective 4, comprehensive experiments on the NEU-DET dataset demonstrate that the improved YOLOv13n achieves mAP50-95 of 0.454 (9.4% relative gain over baseline), mAP50 of 0.774, and mAP75 of 0.466, outperforming mainstream advanced detectors such as FMFFN (mAP50-95 of 0.436) and DynamicTanh (0.434). In terms of computational efficiency, the GFLOPs decrease from 6.2 to 6.1, confirming that the accuracy improvements are achieved without additional computational burden. Generalization tests on GC10-DET show that mAP75 and mAP50-95 improve by 0.033 and 0.019, respectively, while mAP50 remains comparable, validating strong cross-dataset generalization. The proposed module thus effectively balances detection accuracy and lightweight characteristics, providing a high-performance solution for industrial steel defect detection.

To further address the limited multi-scale fusion capability of the Neck structure for tiny defects, future work will investigate the integration of adaptive feature pyramids or attention-guided cross-scale connections to enhance the interplay between shallow spatial details and high-level semantics. In addition, channel pruning will be applied to the A2C2f-MSDE module for structured compression, combined with knowledge distillation to reduce parameters and inference latency without sacrificing accuracy. The generalization robustness under varying illumination and different surface finishing conditions, such as pickling and coating, will also be systematically evaluated.

## Figures and Tables

**Table 1:** Hyperparameter Settings for Model Training.

Hyperparameter	Settings
Input Image Size	640 × 640
Epochs	400
Optimizer	AdamW
Learning Rate	1 × 10^−3^
Momentum	0.937
Weight Decay	0.0005
Learning Rate Scheduling Strategy	Linear Learning Rate Decay
Early Stopping	Patience = 100

**Table 2:** Ablation experimental results of the A2C2f-MSDE module.

Baseline	MSKF-L	SEL	DE	Optimized Residual Structure	mAP50	mAP75	mAP50-95	Parameters	GFLOPs	FPS
YOLOv13n					0.742	0.391	0.415	2.449 M	6.2	1111
	√				0.744	0.443	0.431	2.248 M	6.0	1250
	√	√			0.764	0.412	0.433	2.250 M	6.0	1111
	√	√	√		0.754	0.443	0.438	2.337 M	6.1	1250
	√		√	√	0.756	0.416	0.438	2.334 M	6.1	1111
	√	√		√	0.765	0.416	0.435	2.250 M	6.0	833
	√	√	√	√	0.774	0.466	0.454	2.337 M	6.1	555

**Table 3:** Comparison results with current mainstream object detection models.

	GFLOPs	Precision	Recall	mAP50	mAP75	mAP50-95
YOLOv13n [18]	6.2	0.733	0.697	0.742	0.391	0.415
EDFFN [20]	6.4	0.737	0.698	0.750	0.415	0.432
Spatially-Enhanced Feedforward Network [21]	7.5	0.797	0.701	0.762	0.397	0.425
Mona [22]	6.8	0.805	0.668	0.757	0.420	0.433
FMFFN [23]	5.8	0.750	0.729	0.766	0.426	0.436
DFFN [24]	6.2	0.728	0.721	0.754	0.444	0.428
FRFN [25]	6.5	0.715	0.742	0.750	0.421	0.428
DynamicTanh [26]	6.2	0.771	0.683	0.728	0.447	0.434
SpectralEnhancedFFN [27]	6.5	0.788	0.688	0.753	0.436	0.430
MSDE (Ours)	6.1	0.805	0.713	0.774	0.466	0.454

**Table 4:** Detection results on the NEU-DET dataset and the GC10-DET dataset.

Dataset	Model	GFLOPs	Precision	Recall	mAP50	mAP75	mAP50-95
NEU-DET	YOLOv13n	6.2	0.733	0.697	0.742	0.391	0.415
	MSDE(Ours)	6.1	0.805	0.713	0.774	0.466	0.454
GC10-DET	YOLOv13n	6.2	0.799	0.614	0.702	0.340	0.369
	MSDE(Ours)	6.1	0.710	0.672	0.704	0.373	0.388

## Data Availability

The original contributions presented in this study are included in the article. Further inquiries can be directed to the corresponding author.
